# Systematically Evaluating Cell‐Free DNA Fragmentation Patterns for Cancer Diagnosis and Enhanced Cancer Detection via Integrating Multiple Fragmentation Patterns

**DOI:** 10.1002/advs.202308243

**Published:** 2024-06-17

**Authors:** Yuying Hou, Xiang‐Yu Meng, Xionghui Zhou

**Affiliations:** ^1^ Hubei Key Laboratory of Agricultural Bioinformatics College of Informatics Huazhong Agricultural University Wuhan 430070 China; ^2^ Health Science Center Hubei Minzu University Enshi 445000 China; ^3^ Hubei Provincial Clinical Medical Research Center for Nephropathy Hubei Minzu University Enshi 445000 China; ^4^ Key Laboratory of Smart Farming for Agricultural Animals Ministry of Agriculture and Rural Affairs Wuhan 430070 China

**Keywords:** Cell‐free DNA, early cancer detection, fragmentation patterns, open chromatin regions

## Abstract

Cell‐free DNA (cfDNA) fragmentation patterns have immense potential for early cancer detection. However, the definition of fragmentation varies, ranging from the entire genome to specific genomic regions. These patterns have not been systematically compared, impeding broader research and practical implementation. Here, 1382 plasma cfDNA sequencing samples from 8 cancer types are collected. Considering that cfDNA within open chromatin regions is more susceptible to fragmentation, 10 fragmentation patterns within open chromatin regions as features and employed machine learning techniques to evaluate their performance are examined. All fragmentation patterns demonstrated discernible classification capabilities, with the end motif showing the highest diagnostic value for cross‐validation. Combining cross and independent validation results revealed that fragmentation patterns that incorporated both fragment length and coverage information exhibited robust predictive capacities. Despite their diagnostic potential, the predictive power of these fragmentation patterns is unstable. To address this limitation, an ensemble classifier via integrating all fragmentation patterns is developed, which demonstrated notable improvements in cancer detection and tissue‐of‐origin determination. Further functional bioinformatics investigations on significant feature intervals in the model revealed its impressive ability to identify critical regulatory regions involved in cancer pathogenesis.

## Graphical abstract

Cell‐free DNA within open chromatin regions is susceptible to fragmentation. This study evaluates the diagnostic performance of 10 representative fragmentation patterns in open chromatin regions across 4 datasets for cancer diagnosis. An ensemble model is constructed by combining these fragmentation patterns, demonstrating a more stable performance for cancer detection and tissue‐of‐origin determination. The model's crucial regions offer biologically interpretable insights.

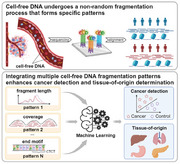

## 1 Introduction

Early detection and diagnosis of cancer are crucial for improving patient survival and treatment outcomes.^[^ ^1^, ^2^, ^3^, ^4^, ^5^ ^]^ Identifying reliable biomarkers is essential for the early detection of cancer.^[^ ^1^, ^3^ ^]^ In this context, liquid biopsies are valuable for assessing circulating biomarkers in bodily fluids and provide informative insights.^[^ ^6^, ^7^, ^8^, ^9^ ^]^ Among these biomarkers, cell‐free DNA (cfDNA) has gained recognition as a promising noninvasive biomarker for liquid biopsies.^[^ ^10^, ^11^ ^]^ It carries genetic and epigenetic alterations specific to cancer, making it a valuable diagnostic tool.^[^ ^5^, ^12^, ^13^, ^14^ ^]^ Most cfDNA in plasma is derived from the hematopoietic system,^[^ ^15^, ^16^, ^17^ ^]^ where nucleated blood cells, such as neutrophils and lymphocytes, are considered primary contributors.^[^ ^18^, ^19^ ^]^ Increasing cfDNA quantities are released into circulation when organs or tissues are affected by pathological conditions.^[^ ^11^, ^20^, ^21^ ^]^ The cfDNA levels observed in patients with cancer are higher than those in healthy individuals, likely because of increased cell death in tumors, which is also a possible marker for tumor aggressiveness.^[^ ^22^, ^23^, ^24^ ^]^ In patients with metastatic cancer, cfDNA levels have been found to be higher than those in patients with localized cancer.^[^ ^25^, ^26^ ^]^ Tumor‐derived cfDNA has a short 1.5–3 h half‐life,^[^ ^27^ ^]^ which facilitates real‐time body monitoring and its application in cancer diagnosis, treatment, and prognosis.^[^ ^26^, ^28^ ^]^ When compared with previously reported blood biomarkers, cfDNA shows higher sensitivity.^[^ ^29^, ^30^ ^]^

The cfDNA undergoes a non‐random fragmentation process that generates a specific pattern, allowing inference of its tissue origin.^[^ ^5^, ^11^, ^31^, ^32^, ^33^ ^]^ It comprises a mixture of DNA fragments of varying sizes, with a prominent peak at approximately 166 bp and a 10‐bp periodicity below 143 bp, which is attributed to nucleosome structure.^[^ ^18^, ^34^, ^35^, ^36^ ^]^ Compared to healthy controls, patients with colorectal cancer exhibited enhanced cfDNA fragmentation.^[^ ^37^ ^]^ with shorter cfDNA lengths originating from the tumor.^[^ ^11^, ^38^, ^39^, ^40^ ^]^ The cfDNA fragmentation pattern refers to information regarding fragment length, distribution, and endpoint sequences in the genome after cfDNA sequencing, which carries numerous signals from tumors.^[^ ^10^, ^41^, ^42^, ^43^ ^]^ Various fragmentation patterns have been proposed and utilized as features to distinguish patients with cancer from healthy individuals, such as windowed protection score (WPS),^[^ ^44^ ^]^ DNA evaluation of fragments for early interception (DELFI),^[^ ^45^ ^]^ and integrated fragmentation score (IFS).^[^ ^10^ ^]^ The cfDNA fragmentation patterns have been widely applied in cancer diagnosis. Classification models constructed using these fragmentation patterns have achieved good classification performance.^[^ ^46^ ^]^ However, the diagnostic performance of most fragmentation patterns has only been assessed in individual datasets and has not been validated using multiple datasets.^[^ ^46^, ^47^ ^]^ Systematically evaluating all fragmentation patterns in large‐scale datasets, which will greatly contribute to subsequent research, is an urgent need.

Considering that cfDNA has several genomic features, some studies have suggested that pan‐cancer detection based on cfDNA should be a multimodal approach utilizing qualitative (i.e., genetic or epigenetic alterations) and quantitative parameters.^[^ ^37^, ^48^ ^]^ Therefore, a few studies have attempted to integrate these diverse features to improve diagnostic model performance.^[^ ^46^ ^]^ For example, Jamshidi et al. evaluated several cfDNA features, including whole‐genome methylation, single nucleotide variants, fragment lengths, somatic copy number variation, allelic imbalance, and fragment endpoints, and constructed an integrated classifier based on these features for multicancer early detection.^[^ ^49^ ^]^ Nguyen et al. improved early multicancer detection using cfDNA methylation and fragment size as features.^[^ ^50^ ^]^ Yaqi Wang et al. found that combining circulating tumor DNA, cfDNA fragment size information, and end motifs could improve early diagnosis of locally advanced rectal cancer.^[^ ^51^ ^]^ Bae et al. integrated genome and epigenome models based on cfDNA to enhance cancer detection.^[^ ^52^ ^]^ These studies combined cfDNA‐derived fragmentation and non‐fragmentation features, in which limited fragmentation patterns were included. Wang et al. confirmed that integrating fragment size ratio (FSR), fragment size distribution (FSD), copy number variation, and end motif preference (EDM) could aid in lung cancer detection.^[^ ^53^ ^]^ However, they did not systematically evaluate the diagnostic efficiency of integrated fragmentation patterns across multiple cancer datasets. Considering this knowledge gap, analyzing the significance of integrating various fragmentation features is important.

Circulating cfDNA fragments associated with nucleosomes indicate that DNA in genome open chromatin regions is susceptible to degradation.^[^ ^44^, ^54^ ^]^ Analyzing cfDNA fragments derived from nucleosome‐depleted promoter regions can provide valuable insights regarding tissue gene expression profiles and estimate the circulating tumor DNA burden.^[^ ^52^, ^55^, ^56^ ^]^ As a binding site for DNA regulatory elements and crucial transcription factors involved in disease progression, the open chromatin region is linked to gene expression.^[^ ^10^, ^43^, ^57^ ^]^ The cfDNA that originates from tissue‐specific open chromatin regions carries rich information that allows inference of cfDNA tissue origin and tumor location prediction.^[^ ^32^ ^]^ Therefore, we confined our analyses to open chromatin regions instead of screening the entire genome. Here, we first systematically evaluated the classification performance of 10 cfDNA fragmentation patterns (fragment length,^[^ ^34^ ^]^ promoter fragmentation entropy (PFE),^[^ ^54^ ^]^ FSR,^[^ ^58^ ^]^ FSD,^[^ ^58^ ^]^ fragment coverage,^[^ ^32^ ^]^ fragment end,^[^ ^59^ ^]^ orientation‐aware cell‐free fragmentation (OCF),^[^ ^32^ ^]^ IFS,^[^ ^10^ ^]^ WPS,^[^ ^44^ ^]^ and EDM.^[^ ^42^ ^]^), in multiple cfDNA sequencing datasets. This indicates that all fragmentation patterns have cancer‐detection capabilities but are unstable. We integrated these fragmentation patterns to construct an ensemble classifier and performed functional annotation and multi‐omics data analyses. These analyses demonstrated that integrating multiple fragmentation patterns enhanced cancer detection model performance and could significantly enrich the regulatory elements implicated in cancer pathogenesis.

## 2 Results

### 2.1 Study Outline

**Figure 1:**
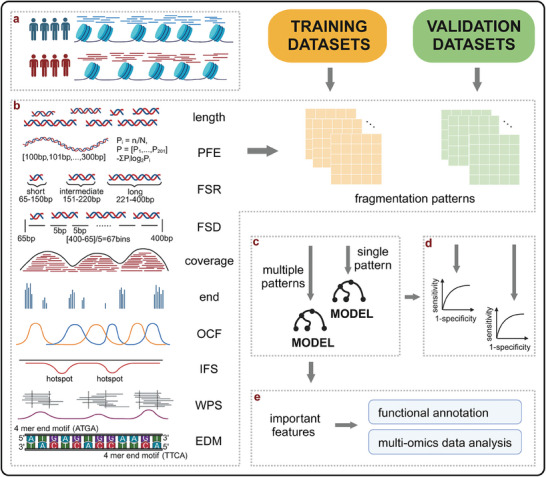
Study workflow. The study involved: a) Collating candidate open chromatin regions. b) Calculating 10 cfDNA fragmentation pattern features for each cfDNA sequencing dataset. c) Constructing cross and independent validation models for single fragmentation patterns or Integrated Fragmentation Patterns. d) Evaluating all model performances. e) Investigating the biological function of important features in the ensemble classifier. PFE: Promoter Fragmentation Entropy; FSR: Fragment Size Ration; FSD: Fragment Size Distribution; OCF: Orientation‐aware Cell‐free Fragmentation; IFS: Integrated Fragmentation Score; WPS: Windowed Protection Score; EDM: End Motif.

The study outline is shown in **Figure** **1**. We compiled open chromatin regions from B cells, T cells, monocytes, and pan‐cancer samples to establish a comprehensive set of these regions. Because of the unavailability of open chromatin data in Roadmap Epigenomics.^[^ ^60^ ^]^ or ENCODE.^[^ ^61^ ^]^ datasets, neutrophils were not included, despite being a major contributor to cfDNA.^[^ ^10^ ^]^ We examined fragmentation patterns from open chromatin regions and assessed their diagnostic performance on plasma cfDNA sequencing datasets from 4 distinct sources. We computed values of 10 published fragmentation patterns for each sample in the collected cfDNA sequencing datasets (see Experimental Section for details). To construct classification models, we employed datasets of Cristiano et al.^[^ ^45^ ^]^ and Jiang et al.^[^ ^59^ ^]^ separately, creating a model for each fragmentation pattern, which was evaluated using cross‐validation. For the Cristiano et al. dataset, all cancers jointly constructed pan‐cancer classifiers and individually constructed cancer‐specific classifiers were included. External validation was performed using independent datasets from Zhou et al.^[^ ^10^ ^]^ and Mathios et al.^[^ ^62^ ^]^ For each training dataset, we integrated 10 fragmentation patterns to construct an ensemble classifier, defined as “Integrated Fragmentation Pattern (IFP),” with similar methods for validation and evaluation (see Experimental Section). We functionally analyzed essential features of the ensemble model using functional annotation and omics data analyses for biological interpretability.

### 2.2 Performance Comparisons of cfDNA Fragmentation Patterns for Cancer Diagnosis

**Figure 2:**
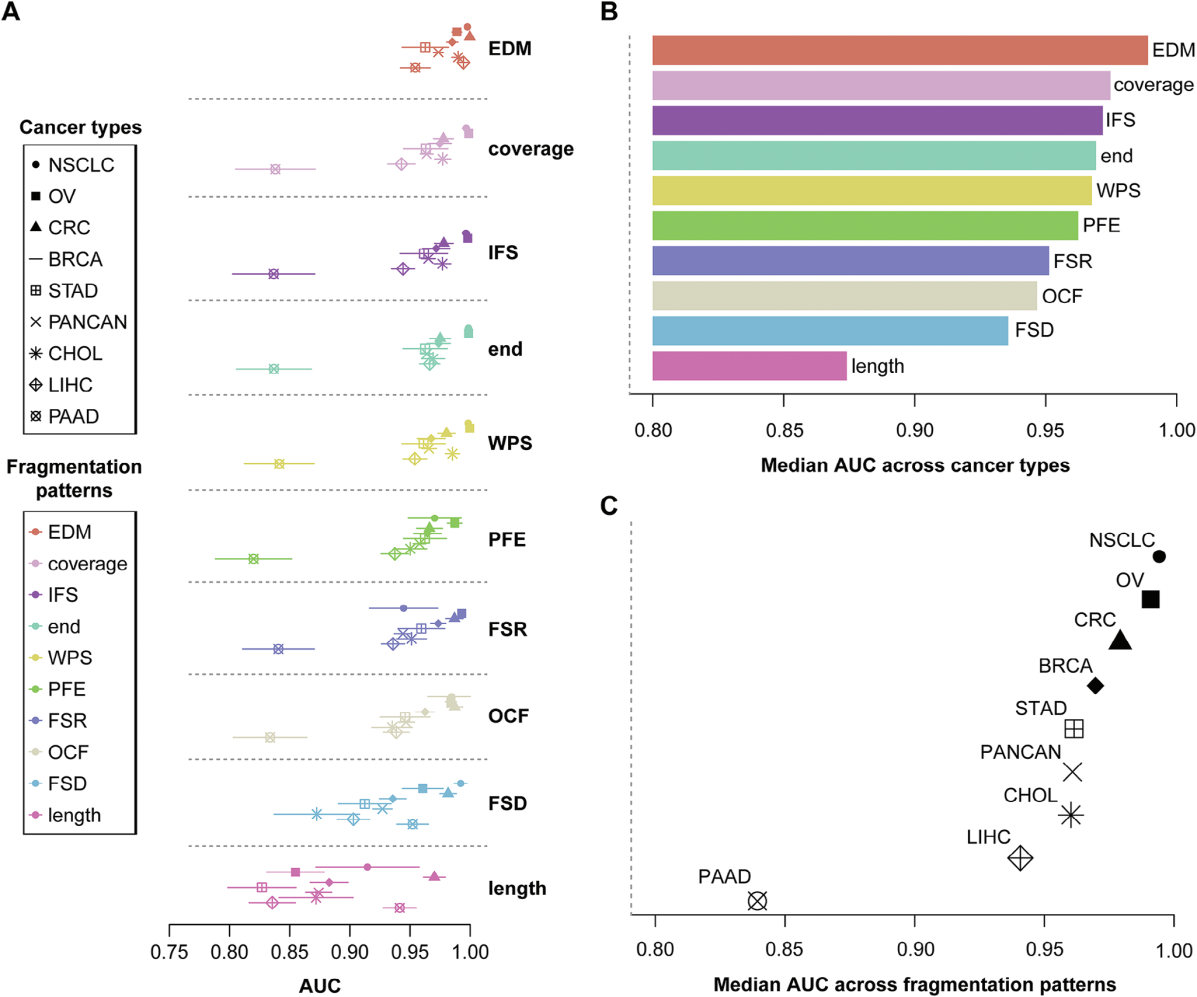
Performance of 10 cfDNA fragmentation patterns using cross‐validation in the Cristiano et al. and Jiang et al. datasets. A) Performance of all cfDNA fragmentation patterns across all cancer types (AUC). Data is presented as means and 95% confidence intervals. B) Ranking of median values of each cfDNA fragmentation pattern across all cancer types (AUC). C) Ranking of median values of each cancer type across all cfDNA fragmentation patterns (AUC). PFE: Promoter Fragmentation Entropy; FSR: Fragment Size Ration; FSD: Fragment Size Distribution; OCF: Orientation‐aware Cell‐free Fragmentation; IFS: Integrated Fragmentation Score; WPS: Windowed Protection Score; EDM: End Motif; PANCAN: Pan‐cancer; BRCA: breast cancer; CHOL: cholangiocarcinoma; CRC: colorectal cancer; STAD: gastric cancer; NSCLC: lung cancer; OV: ovarian cancer; PAAD: pancreatic cancer; LIHC: liver cancer; AUC: area under the receiver operating characteristic curve.

The 10 selected fragmentation patterns can be divided into 4 categories: fragmentation patterns that use i) fragment length information, such as length, PFE, FSR, and FSD; ii) fragment number (coverage) information, such as fragment coverage (number of fragment midpoints), fragment endpoint number; iii) both fragment lengths and coverage information, such as OCF, IFS, and WPS; and iv) fragment sequence information, such as EDM (see Experimental Section). Results of these patterns on all datasets are shown in **Figure** **2** and Figure S1 (detailed results: Tables S2 and S3, Supporting Information).

All fragmentation patterns showed good diagnostic performances (Figure 2A,B; Figure S1A,B, Supporting Information). Classification using only length features demonstrated slightly inferior accuracy, whereas using only the fragment number showed better classification results. Fragmentation patterns that considered both fragment length and number performed well, suggesting that the number of fragment features was sufficient to capture considerable differential signals and that combining them with other information could provide benefits. The fragmentation pattern with base resolution demonstrated the best performance (EDM), indicating that exploring specific base types in cfDNA fragments in addition to the genome cfDNA distribution, warrants further investigation. We observed that EDM performance was not sufficiently stable in independent validation. Conversely, OCF, IFS, and WPS, which involved both coverage and length information, generalized better (Table S3, Supporting Information). The correlation between cfDNA fragmentation patterns was analyzed from 2 perspectives (see Experimental Section). Correlation analysis of predicted probabilities associated with these fragmentation patterns (Figure S2A, Supporting Information) revealed a strong correlation between PFE, coverage, end, IFS, and WPS, suggesting that these patterns generally yield similar classification results, likely because they are distinctly associated with nucleosome positioning information. Conversely, the length, FSD, and EDM exhibited lower correlations with other fragmentation patterns. A significant positive correlation was observed among PFE, coverage, end, and IFS, whereas WPS displayed a notably negative correlation (Figure S2B, Supporting Information). As mentioned previously, PFE, coverage, end, and IFS are intricately linked to nucleosome‐depleted positions, whereas WPS characterizes nucleosome‐occupied position information; they underscore the pronounced correlation between nucleosome positioning information and specific fragmentation patterns, whereas correlations with other pattern types remain relatively modest.

We comprehensively compared diagnostic results of various cancer types (Figure 2A,C; Figure S1A,C, Supporting Information). These findings indicated that models for lung cancer, ovarian cancer (OV), and colorectal cancer exhibited superior diagnostic performance. Conversely, the pancreatic cancer (PAAD) model demonstrated the lowest diagnostic performance, possibly because PAAD generally had low cfDNA content.^[^ ^63^ ^]^ Despite the generally poor performance of length as a fragment feature in most cancer types, it performed relatively better in PAAD compared to other fragmentation patterns.

### 2.3 Classification Performance of IFP

**Figure 3:**
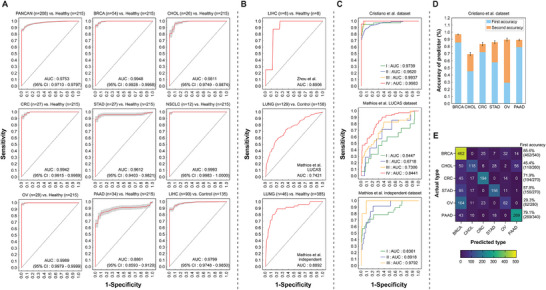
Performance of Integrated Fragmentation Pattern (IFP) for cancer detection and tissue‐of‐origin determination. A) Cross‐validation results for IFP. B) Independent validation results for IFP. C) IFP performance in different cancer stages. Cristiano et al. dataset: I (*n* = 41), II (*n* = 109), III (*n* = 33), IV (*n* = 22); Mathios et al. LUCAS dataset: I (*n* = 15), II (*n* = 7), III (*n* = 35), IV (*n* = 72); Mathios et al. independent dataset: I (*n* = 28), II (*n* = 12), III (*n* = 5). D) The first and second accuracy for each cancer in the multiclass model in the Cristiano et al. dataset (excluding lung cancer). (E) The actual and first‐ranked predicted types for each cancer in the multiclass model (excluding lung cancer). Values presented in the table represent the total samples used in the 10 times 10‐fold cross‐validation. First accuracy: Accuracy calculated according to the top‐ranked prediction category in the classifier. Second accuracy: Accuracy is calculated according to the second‐ranked prediction category in the classifier. Data is presented as means and 95% confidence intervals. The curve shows the mean and the shadow shows the 95% confidence interval range. PANCAN: Pan‐cancer; BRCA: breast cancer; CHOL: cholangiocarcinoma; CRC: colorectal cancer; STAD: gastric cancer; NSCLC: lung cancer; OV: ovarian cancer; PAAD: pancreatic cancer; LIHC: liver cancer; LUNG: lung cancer.

We compared the performance of IFP with other fragmentation patterns using cross and independent validations and found that IFP exhibited superior classification performance and demonstrated greater stability (Figure S3, Supporting Information). IFP exhibited good classification performance in detecting all 8 cancer types, including pan‐cancer diagnosis. The area under the receiver operating characteristic curve (AUC) values for all classifiers were > 0.90, except for PAAD (AUC = 0.8861), indicating satisfactory performance (**Figure** **3**A). We validated ensemble classifiers for liver and lung cancers using 3 independent datasets (one and 2 for liver and lung cancers, respectively).^[^ ^10^, ^62^ ^]^. Both the ensemble classifiers performed well during independent validation, supporting their generalizability and stability (Figure 3B).

We assessed the diagnostic performance (AUC) of the IFP model on patients with different cancer stages in 3 datasets with known stage information. In the Cristiano et al. dataset.^[^ ^45^ ^]^ (pan‐cancer versus healthy), classification performance for early‐stage samples was excellent and increased with cancer stage progression (Figure 3C, top). Mathios et al. LUCAS dataset.^[^ ^62^ ^]^ contained a mix of various disease information, because of which its independent validation performance was relatively poor, particularly in the early stages, and showed a moderate diagnostic performance, with an AUC of 0.8441 in stage IV (Figure 3C, middle). In contrast, the Mathios et al. independent dataset.^[^ ^62^ ^]^ exhibited good independent validation performance, achieving impressive classification results even in stage I (AUC = 0.8361) and II (AUC = 0.8918), and an AUC of 0.9792 in stage III (Figure 3C, bottom). This result indicates that IFP can achieve good results across different cancer stages.

Next, we assessed the integrated classifier efficacy in accurately localizing different cancer types. We developed a multi‐label integrated classifier using cancer samples from the Cristiano et al. dataset.^[^ ^45^ ^]^ To avoid smaller data affecting the model, we excluded lung cancer samples (*n* = 12) (refer to Figure S5, Supporting Information for full classification results). Overall, our model achieved a good performance, with a median value of 0.6150 for the first accuracy (Figure 3E, see Experimental Section). The second accuracy exceeded 0.80 for almost all cancers, with a median value of 0.8571 (Figure 3D). Different cancer types showed varying accuracy rates, with breast cancer (BRCA) and PAAD achieving higher accuracy rates, whereas OV had the lowest top 1 accuracy. Nevertheless, in the top 2 accuracy, the OV rate could reach 0.90 (Figure 3D,E; Figure S4, Supporting Information).

### 2.4 IFP Score for Distinguishing Different Sample Categories

**Figure 4:**
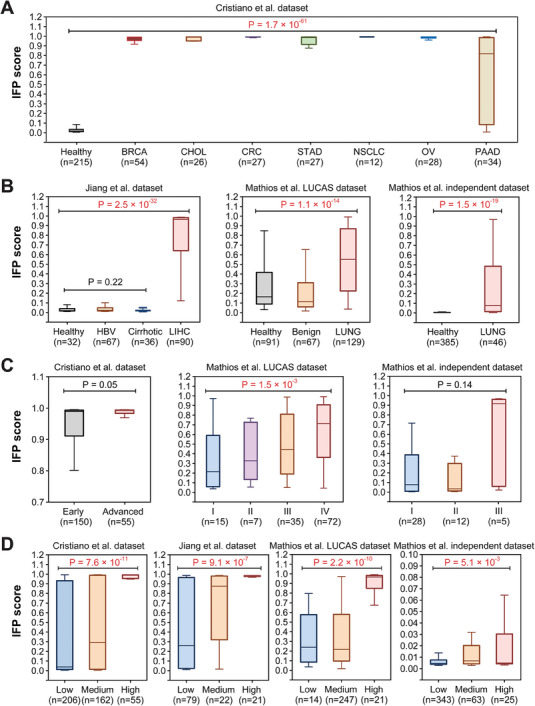
Differences in IFP scores among samples of different categories. A, B) IFP scores across different sample types. C) IFP scores among different cancer stage samples. D) IFP scores among patients with different tumor DNA fractions: Low, [0‐2%]; Medium, [2‐15%]; High, [15‐100%]. Wilcoxon rank‐sum test was applied for the differential analysis of 2 data groups (unpaired). The Kruskal‐Wallis test was used for the differential analysis of multiple data groups (unpaired). IFP: Integrated Fragmentation Pattern; BRCA: breast cancer; CHOL: cholangiocarcinoma; CRC: colorectal cancer; STAD: gastric cancer; NSCLC: lung cancer; OV: ovarian cancer; PAAD: pancreatic cancer; HBV: Hepatitis B; LIHC: liver cancer; LUNG: lung cancer.

To construct the integrated classifier, we assigned the predictive probability provided by it to each sample as the IFP score; a score closer to 1 indicated a higher likelihood of the sample being classified as cancerous. Significant differences were observed in IFP values among various sample types (**Figure** **4**A,B). In the Jiang et al. dataset,^[^ ^59^ ^]^ patients with liver cancer exhibited significantly higher IFP scores than those with liver cirrhosis, hepatitis B, and healthy individuals, whereas no significant differences were found among the 3 noncancer sample types (Figure 4B, left). A similar pattern was observed for Mathios et al. LUCAS dataset,^[^ ^62^ ^]^ where patients with cancer showed distinct differences compared to those without cancer, whereas benign and healthy samples did not show clear differentiation (Figure 4B, middle). Another lung cancer dataset from Mathios et al. also demonstrated significant differences in IFP values between patients with lung cancer and healthy samples (Figure 4B, right). Collectively, these findings demonstrate that IFP values can effectively distinguish patients with cancer from other samples, further emphasizing its utility in cancer diagnosis.

For stage‐stratified analyses (Figure 4C), patients with late‐stage cancer exhibited higher IFP values than those with early‐stage cancer. We used “ichorCNA”.^[^ ^64^ ^]^ to estimate the tumor DNA fraction of each sample and categorized samples into low [0%–2%], medium [2%–15%], and high [15%–100%] based on the fraction. Significant differences were observed in IFP scores of samples with different tumor DNA fractions (Figure 4D).

Overall, these results indicate the significance of IFP scores in cancer diagnosis.

### 2.5 Biological Implications of Critical Regions in the Ensemble Classifier

**Figure 5:**
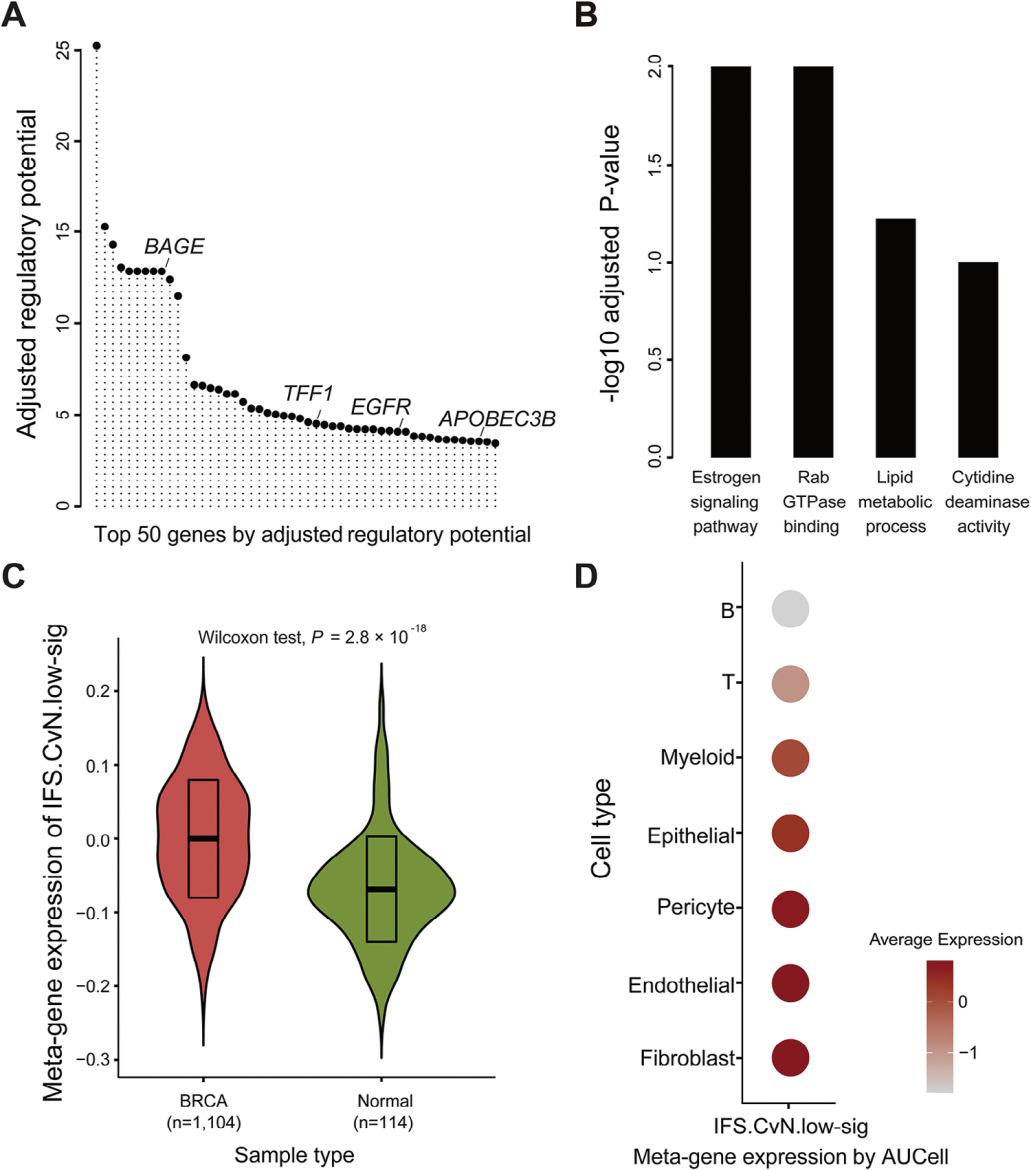
Biological exploration of the top 15k critical regions in the breast cancer model. A) Top 50 genes by adjusted regulatory potential revealed using gene‐mapping with Cistrome‐GO enhance mode. B) Significantly enriched functional ontologies associated with the top 15k regions. C) Meta‐gene expression of the IFS.CvN.low‐sig (1431 genes) in log2 RSEM RNA‐seq transcriptomes of breast cancer tumors versus adjacent normal tissues. D) Cell‐type‐specific meta‐gene expression of IFS.CvN.low‐sig (1431 genes). Wilcoxon test: Wilcoxon rank‐sum test. IFS: Integrated Fragmentation Score; BRCA: breast cancer.

Critical regions in the ensemble classifier were studied using BRCA as the subject of interest, because it has a high burden of disease, and noninvasive detection methods are urgently needed. Based on the model built with the Cristiano et al. dataset for BRCA (*n* = 54) versus non‐cancer controls (*n* = 215), we identified a set of critical regions defined as regions with the top 15k contribution significance to model classification (Experimental Section). Through Cistrome‐GO analysis under enhancer mode, (within the distance 15 × 10 kb),^[^ ^65^ ^]^ top genes mapped to these regions (genes overlapping [‐75 kb, 75 kb] as corresponding top genes) were found to include estrogen‐regulated BRCA genes (eg., *TFF1*), cancer/testis antigens (eg., *BAGE*), epithelial growth factor receptor (eg., *EGFR*), and genes involved in cancer mutagenic process (eg., *APOBEC3B*)^[^ ^66^, ^67^, ^68^, ^69^ ^]^ (**Figure** **5**A; Table S4, Supporting Information). These regions were significantly enriched for BRCA‐related functional ontologies, including the estrogen signaling pathway (hsa04915, FDR = 0.01), Rab GTPase binding (GO:00 17137, FDR = 0.01), lipid metabolic processes (GO:0 006629, FDR = 0.06), and cytidine deaminase activity (GO:0 004126, FDR = 0.1).^[^ ^68^, ^70^, ^71^, ^72^ ^]^ (Figure 5B; Table S5, Supporting Information).

We investigated the ability of critical regions to capture gene expression deregulation programs in BRCA tumors. We created a signature of genes mapped to regions with significantly lower IFS scores in patients with BRCA than in controls, named IFS.CvN.low‐sig (Table S6, Supporting Information). IFS score and chromatin openness are negatively associated; hence, meta‐gene expression of this signature was expected to be upregulated in BRCA tumors compared to that in adjacent normal tissues.^[^ ^10^ ^]^ Using The Cancer Genome Atlas (TCGA) BRCA data and Gene Set Variation Analysis (GSVA), we showed a significantly higher meta‐gene expression level of IFS‐sig in tumors (*n* = 1104) than in adjacent normal samples (*n* = 114), suggesting that tumor‐altered gene expression profiles were captured by critical regions (Wilcoxon rank‐sum test, *P* = 2.8 × 10^−18^; Figure 5C). We further dissected the cellular origins of signature genes using single‐cell RNA‐seq analysis. A total of 96088 cells (epithelial, 25618; fibroblast, 6469; endothelial, 7412; pericyte, 5312; myeloid, 9562; T, 35053; B, 6662) collected from 26 BRCA samples were analyzed. Tumor stroma, including fibroblast and tumor vasculature, showed the highest expression of signature genes, followed by the epithelial cancer cells, whereas expression was low in immune cells, particularly in lymphocytes. This observation suggests an important contribution of stromal remodeling in cfDNA fragmentomics‐based non‐invasive detection of BRCA (Figure 5D).

## 3 Discussion

Recently, cfDNA has emerged as a promising biomarker for cancer diagnosis, organ transplant monitoring, and noninvasive prenatal testing.^[^ ^11^, ^21^, ^73^ ^]^ In early cancer diagnosis, several fragmentation patterns have been proposed and validated for diagnostic performance;^[^ ^42^, ^45^, ^59^ ^]^ however, most have only been validated on their own or on limited datasets, making the specific performance of each fragmentation pattern uncertain. This can significantly affect their practical applications. Therefore, we conducted a comprehensive evaluation of 10 cfDNA fragmentation patterns via collecting plasma cfDNA sequencing data from 4 different sources encompassing 8 cancer types. Some fragmentation patterns are defined based on position, length, or sequence information of fragments spanning the entire genome or large genomic regions. Others are derived from relevant information specific to active regulatory regions. Larger fragmentation patterns that span broad regions lack biological interpretability as they cannot be isolated to specific regulatory regions.^[^ ^10^, ^45^, ^58^ ^]^ Conversely, alterations in fragmentation patterns within open chromatin regions are known to exhibit superior efficacy for cancer diagnosis.^[^ ^10^, ^54^, ^55^ ^]^ Therefore, we analyzed open chromatin regions specific to the hematopoietic system of blood cells and pan‐cancer to characterize cfDNA fragmentation patterns with greater relevance and specificity.

The results demonstrate the cancer diagnostic capabilities of all fragmentation patterns. Those based solely on fragment length showed slightly poorer results, inferior to those considering both fragment count and size information. EDM utilizes fragment endpoint sequence information; it showed a superior performance. The performance of the fragmentation patterns combining fragment length and number information was basically more stable in independent validation. Within each category, those with more precise characterization exhibited better performance. For instance, directly using fragment length (fragment lengths spaced at 10‐bp intervals) yielded the worst results. However, a notable improvement was observed when we incorporated fragment length information as a feature for each open chromatin region (PFE and FSR), with PFE displaying the best performance. This significant enhancement may be because the PFE method was used, which characterized the ratio of fragments at different length intervals using length Shannon entropy.^[^ ^54^ ^]^ This approach increased precision in describing fragment size variation in open chromatin regions, resulting in better predictive capabilities. Therefore, among the 4 fragmentation pattern categories, those with comprehensive or precise information tended to perform better. Further validation of this discovery using additional datasets is required. Despite confirming the diagnostic value of all fragmentation patterns, their performance varied significantly across different cancer types and datasets. Fragmentation patterns exhibit better diagnostic performance for colorectal, ovarian, and lung cancers, whereas that for PAAD is significantly poorer. Features such as length and FSD, which typically perform poorly, show better results for PAAD than other fragmentation patterns. Considering validation results from independent datasets, the fragmentation pattern performance showed a more significant variation. Validating the same lung cancer classifier with 2 independent datasets yielded significantly different classification outcomes, indicating dataset nature significantly affected classification results. Collectively, these findings suggest a lack of diagnostic stability in fragmentation patterns.

We integrated all 10 fragmentation patterns to construct an ensemble classifier. IFP exhibited higher and more stable classification capabilities in both cross and independent validations. IFP demonstrated good predictive accuracy for tissue‐of‐origin determination. Most misclassified OV samples were predicted to be BRCA (Figure 3E), possibly because of the high similarity in their cfDNA fragmentation patterns. Similar phenomena have been observed in other studies,^[^ ^74^ ^]^ where misclassified samples are often assigned to highly related tissues; for example, gastric cancer is frequently misclassified as colorectal cancer. These results may indicate distinctive information embedded within different cfDNA fragmentation patterns and demonstrate substantial improvement in classification performance via their integration. Although further data validation is needed, this perspective offers a potential direction for using cfDNA fragmentation patterns in cancer diagnosis.

We focused on fragmentation patterns within open chromatin regions; hence, prominent features within our models may be key regulatory regions in cancer development. Through gene mapping and functional annotation, we identified key enriched genes and pathways associated with BRCA. Joint analysis with tumor transcriptomics further confirmed the biological interpretability of these critical regions. These findings provide compelling evidence that our method not only delivers accurate diagnostics but also identifies essential regulatory regions.

Previous studies established that fragmentation patterns near transcription start sites (TSS) can predict gene expression levels and have significant diagnostic value in cancer detection.^[^ ^54^, ^55^ ^]^ We expanded our analysis beyond open chromatin regions and incorporated TSS‐adjacent regions as feature regions. Our findings mirrored those observed in open chromatin regions, with those displaying a marginally superior overall performance compared to TSS regions (Tables S7 and S8, Supporting Information). A support vector machine (SVM) and alternative classifier models, including Random Forest (RF), Naïve Bayes (NB), Logistic Regression (LR), and XGBoost were employed and detailed results are presented in Figure S6 and Table S9 (Supporting Information). Different models yielded similar conclusions, but SVM showed the best performance overall; in conjunction with previous research,^[^ ^10^ ^]^ SVM was selected as the primary classifier model. After correcting the potential influence of GC bias in the cfDNA data, the results in Table S10 (Supporting Information) validate our findings. The process of dataset collection, sequencing platforms, and data handling methods may introduce biases between datasets, thereby influencing the performance of the model. Currently, no batch‐effect correction tool specifically designed for cfDNA fragmentation patterns is available; we directly only used the Z‐score to standardize each sample during model development and validation. To examine the benefit of batch correction tools on classifier performance on independent validation, we also utilized the most widely used tool, “combat,” to correct data from different sources.^[^ ^75^ ^]^ After batch‐effect removal, the classifier performance remained consistent with that before removal (Table S11, Supporting Information), possibly because the tool was not specifically designed for cfDNA fragmentation patterns. Considering that feature dimensions of different fragmentation patterns vary considerably, we performed a principal component analysis dimensionality reduction on all fragmentation patterns to the same dimensions before constructing the classification model. Model performances before and after dimensionality reduction were found to be similar (**Table** **1**). Our use of fragmentation patterns within open chromatin regions partially changed the original definition; hence, we recalculated fragmentation patterns originally not defined within specific regulatory regions and constructed a classification model based on the original definition.^[^ ^42^, ^45^, ^58^ ^]^ Fragment length and EDM were calculated for the whole genome, but FSR and DELFI divided the whole genome into 5 mb bins; whereas the FSD definition was based on each chromosome arm. These adjustments changed the fragmentation pattern diagnostic performance, slightly increasing the effectiveness of fragment length, FSD, and EDM, and decreasing FSR performance (Table 1). However, these modifications did not affect the conclusions from our previous evaluations, which indicated that EDM exhibited the highest performance in cross‐validation, followed by fragmentation patterns that incorporated both fragment length and coverage information. DELFI calculates the proportion of short and long fragments in large regions, such as 5 mb, which is similar to FSR. Our work mainly focused on evaluating fragmentation patterns in genomic regulatory regions; hence, we opted not to include DELFI in the Results section. However, its diagnostic performance was found to be excellent, with an AUC of 0.9575, slightly trailing behind EDM, IFS, WPS, and coverage (Table 1). Moreover, we have also analyzed the classification performance of incorporating DELFI into IFP, revealing that DELFI slightly enhances IFP's performance (Tables S12 and S13, Supporting Information). To remove redundant information from IFP, we used “mRMR” for feature filtering.^[^ ^76^ ^]^ Feature filtering was performed to select important features (fragmentation pattern predictions) and then train the ensemble classifier again. Overall, classifier performance progressively improved as the number of features increased; however, it was still weaker than that of the classifier that used all features. Despite the improved classifier performance, we used all fragmentation patterns in the IFP to combine all results (Tables S14 and S15, Supporting Information). The reliability of our findings can be substantiated through comparisons across various scenarios.

**Table 1:** Comparison of cfDNA fragmentation patterns across various scenarios (AUC).

	open chromatin region	after PCA degradation	definition as per the original publication
length	0.8741 (0.8634–0.8848)	0.8763 (0.8661–0.8865)	whole genome 0.8835 (0.8739–0.8930)
PFE	0.9579 (0.9528–0.9631)	0.9577 (0.9523–0.9631)	/
FSR	0.9441 (0.9369–0.9513)	0.9447 (0.9389–0.9505)	5mb bins 0.8456 (0.8344–0.8568)
FSD	0.9271 (0.9191–0.9351)	0.9229 (0.9149–0.9308)	chromosome arm 0.9431 (0.9365–0.9498)
coverage	0.9638 (0.9585–0.9692)	0.9640 (0.9595–0.9684)	/
end	0.9639 (0.9586–0.9692)	0.9625 (0.9573–0.9676)	/
OCF	0.9467 (0.9399–0.9536)	0.9469 (0.9401–0.9538)	/
IFS	0.9653 (0.9595–0.9710)	0.9641 (0.9592–0.9690)	/
WPS	0.9658 (0.9598–0.9719)	0.9649 (0.9596–0.9701)	/
EDM	0.9736 (0.9696–0.9776)	0.9737 (0.9695–0.9780)	whole genome 0.9824 (0.9786–0.9862)
DELFI	/	/	5 mb bins 0.9575 (0.9520–0.9631)

Our study had some limitations. First, although we attempted to collect all available cfDNA datasets, the overall sample size was not sufficient. Expanding sample sizes in future studies may enhance statistical power and provide more robust conclusions. Second, we used data from next‐generation sequencing; therefore, we focused on short cfDNA fragments (< 500 bp in length). However, with advances in sequencing technology, single‐molecule sequencing (third‐generation sequencing) has revealed numerous long cfDNA molecules up to several kilobases in the plasma DNA of healthy individuals and patients with cancer.^[^ ^77^, ^78^, ^79^ ^]^ These molecules have been demonstrated to be preferentially derived from open chromatin regions and their abundance has been correlated with transcriptional activities.^[^ ^80^ ^]^ We did not include studies on long cfDNA fragments due to limitations in short‐read of next‐generation sequencing techniques. These aspects warrant further investigation and we are committed to exploring them in future research endeavors.

In summary, we comprehensively evaluate 10 previously published fragmentation patterns for early cancer detection. Our study highlights the diagnostic potential inherent in all 10 patterns, particularly noting that those containing richer and more precise information demonstrate superior diagnostic performance. However, we also observe a limitation in the stability of these patterns across different datasets. To address this challenge, we propose an integrated approach that capitalizes on the strengths of this pattern. Through integration, we achieve enhanced performance in the diagnosis of specific cancers, pan‐cancer diagnosis, and tissue‐of‐origin determination. Importantly, our ensemble model exhibits biological interpretability, supported by the analysis of biologically relevant features. Beyond pattern evaluation, our work provides valuable insights for the development of improved fragmentation patterns. Furthermore, the robust performance and biological interpretability of our ensemble model provide crucial references for future research in this field.

## 4 Experimental Section

### Cell‐Free DNA Whole Genome Sequencing Data and Preprocessing

Details of cfDNA sequencing datasets collected from 4 different sources are shown in Table S1 (Supporting Information). The Cristiano et al. dataset^[^ ^45^ ^]^ consisted of 423 samples, including 215 healthy, 54 BRCA, 26 cholangiocarcinoma, 27 colorectal cancer, 27 gastric cancer, 12 lung cancer, 28 OV, and 34 PAAD samples, with cfDNA fragment information from FinaleDB.^[^ ^81^ ^]^ The Jiang et al. dataset^[^ ^59^ ^]^ contained 225 samples, including 32 healthy, 67 hepatitis B, 36 cirrhotic, and 90 liver cancer samples, with cfDNA fragment information from FinaleDB.^[^ ^81^ ^]^ The Zhou et al. dataset included 8 healthy individuals and 8 liver cancer samples, fragment information was obtained from the original paper.^[^ ^10^ ^]^ Mathios et al. LUCAS dataset had 287 samples, with 91 healthy, 67 benign, and 129 lung cancer samples. The Mathios et al. independent dataset had 431 samples, including 385 healthy and 46 lung cancer samples.^[^ ^62^ ^]^ The GRCh37 human reference genome was used for all data.

For both Mathios et al. datasets, the BAM files of each sample were downloaded from EGA (EGAD00001007796). The data was preprocessed by removing duplicate polymerase chain reaction fragments using Sambamba (v0.8.2).^[^ ^82^ ^]^ Low‐quality fragments were removed using Samtools (v1.3.1) with the following parameters: ‐f 3 ‐F 3852 ‐q 30.^[^ ^83^ ^]^ Only high‐quality reads were retained for all downstream analyses.

### Collection of Feature Regions

Open chromatin regions of B cells, T cells, and monocytes were obtained from the DNA‐seq peak data (broad peaks) from the Roadmap project^[^ ^60^ ^]^ (E29, E32, and E34; Table S1, Supporting Information). Open chromatin regions of patients with pan‐cancer were obtained from the ATAC‐seq peaks (Table S1, Supporting Information).^[^ ^84^ ^]^ For ATAC peaks, the original data were based on the GRCh38 reference genome, which was lifted‐over to the GRCh37 human reference genome. The union set of open chromatin regions was collated using Bedtools (v2.22.0).^[^ ^85^ ^]^ resulting in 561414 candidate open chromatin regions for the 22 autosomes. The centroid of each candidate region was then determined and the length of each region was adjusted to 200 bp by extending 100 bp upstream and downstream of the centroid.

For comparison, the TSS flanking regions were used as feature regions. TSS of the GRCh37 human reference genome was obtained from GENCODE (Table S1, Supporting Information).^[^ ^86^ ^]^ Two different length intervals were used for TSS: (−150 bp, +50 bp) and (−1000 bp, +1000 bp).

### Calculating the Feature Matrix of cfDNA Fragmentation Patterns

In this study, 10 fragmentation patterns were used. According to their definitions, the calculations were adjusted to align with the selected open chromatin regions.

Fragments whose centroids fell within open chromatin regions were first selected.^[^ ^34^ ^]^ For each autosome, fragments shorter than 300 bp were divided into 30 categories at 10 bp intervals, whereas those longer than 300 bp were grouped into a single category. Consequently, a feature vector of length 31 × 22 was generated for each sample.

To calculate PFE,^[^ ^54^ ^]^ after tallying the selected fragments, they were categorized as shorter than 100 bp, and longer than 250 bp, and those ranging from 100–250 bp were further divided into 15 categories at 10 bp intervals. Fragment proportions in each category were computed and the entropy value for that specific region was calculated using the following formula:

(1) $$PFE=-\sum P_{i}log_{2}P_{i}$$

Where *P_i_* is the ratio of fragments in the *i*th category. Hence, 561414 features were calculated for each sample.

To calculate FSR,^[^ ^58^ ^]^ selected fragments were tallied and divided into 3 categories based on their lengths: short (65–150 bp), medium (151–220 bp), and long (221–400 bp). The fragment proportion in each category was calculated and each sample had 561414 × 3 features.

To calculate FSD,^[^ ^58^ ^]^ all selected fragments were tallied. Those with lengths ranging from 65 to 400 bp were divided into 67 categories at 5 bp intervals, and the fragment proportions in each category were calculated. Therefore, each sample contained 67 × 22 features.

For coverage calculation,^[^ ^32^ ^]^ selected fragments were tallied. For each sample, the features consisted of a vector of size 561414 × 1 (for subsequent fragmentation patterns, the number of features for each sample was 561414 × 1 unless otherwise specified).

For fragment ends,^[^ ^59^ ^]^ the number of fragment ends within each open chromatin region was counted.

To calculate OCF,^[^ ^32^ ^]^ coordinates of the position with the smaller fragment genome (U) and that with the larger fragment genome (D) on each chromosome were determined with the corresponding counts. For each open chromatin region, a 20 bp range was selected based on a 60 bp shift upstream and downstream of the centroid. The OCF of the region was calculated using the following equation:

(2) $$OCF=\sum_{-60-10}^{-60+10}\left(D-U\right)+\sum_{60-10}^{60+10}\left(U-D\right)$$

For IFS,^[^ ^10^ ^]^ the fragments (n) within each open chromatin region including the fragment centroid were counted and their average length (l) within the region was calculated. L represents the average fragment length of the whole chromosome. The calculation formula is as follows:

(3) $$IFS=n*\left(1+\frac{l}{L}\right)$$

For WPS,^[^ ^44^ ^]^ according to the genomic coordinate position of each cfDNA fragment, a window of 120 bp was slid at 1 bp intervals, and the likelihood of each base pair being covered at the whole genome level, fully covered (+1), and partially covered (−1), was counted. The mean value of all loci within each open chromatin region was calculated.

For EDM,^[^ ^42^ ^]^ the EDM consisting of 4 bases at the 5′ end of the cfDNA fragment whose fragment centroid was within the open chromatin region was viewed. The proportion of each EDM on each chromosome was counted separately. Therefore, for each sample, the feature had a vector of size 256 × 22.

After the feature matrix was computed, it was standardized with a Z‐score using the “preprocessing. scale” function from the “sklearn” library in Python. To mitigate bias from sequence GC content variations, the GC% covariates were regressed from the original fragmentation pattern scores of each open chromatin region using locally weighted smoothing linear regression (Lowess) with a span of 0.75.^[^ ^10^, ^45^ ^]^

### Classification Model Construction

In the training set, classifier models were constructed using an SVM based on features extracted from each fragmentation pattern and their corresponding class labels for diagnostic tasks. RF, NB, LR, and XGBoost models were included for comparative analysis. All models were built using the “sklearn” library (for XGBoost, “xgboost” library) in Python with default parameters. The training set was cross‐validated to explore optimal parameters; however, the results did not change significantly (data not shown). Hence, default parameters were utilized. For feature dimensionality reduction, the “PCA” function in the “sklearn. decomposition” library was used.

An ensemble classifier was constructed by combining the 10 cfDNA fragmentation patterns. Classification models were built using an SVM for each training dataset, with each fragmentation pattern predicting probabilities for each sample. These probabilities formed a matrix with 10 values per sample, which served as features for constructing the classifier again using the SVM, ultimately determining the final classifier performance. The integrated classifier was then validated using test datasets specific to the corresponding cancer types.

A tissue‐of‐origin analysis was conducted using 7 cancer samples from the Cristiano et al. dataset. A multi‐label classifier based on SVM (function “OneVsRestClassifier” in library “sklearn.multiclass”) was employed to train the organ localization model, using the same ensemble strategy as described previously. Classifier performance was evaluated by considering the first and second‐ranked prediction categories as the first and second accuracies for each sample, respectively.

### Classification Model Evaluation

The pan‐cancer and specific cancer diagnostic models for both the Cristiano et al.^[^ ^45^ ^]^ and Jiang et al.^[^ ^59^ ^]^ datasets and the tissue‐of‐origin analysis were evaluated using 10 times 10‐fold cross‐validation. During independent validation, the liver cancer diagnostic model was built using all samples from Jiang et al.^[^ ^59^ ^]^ dataset and validated using the liver cancer dataset from Zhou et al.^[^ ^10^ ^]^ Similarly, to construct the lung cancer diagnostic model, lung cancer samples and corresponding controls from the Cristiano et al. dataset were used and validated using the 2 lung cancer datasets from Mathios et al.^[^ ^62^ ^]^

For cancer diagnosis, AUC and sensitivity at high specificity levels, including sensitivity at 95% and 85% specificity were the primary evaluation metrics used. For the tissue‐of‐origin analysis, accuracy was the evaluation metric.

### Correlation Analysis of cfDNA Fragmentation Patterns

The correlation between cfDNA fragmentation patterns was analyzed using 2 methods. 1) Cancer diagnostic models were constructed using each cfDNA fragmentation pattern separately from the Cristiano et al. dataset. Spearman's correlation analysis was performed on sample prediction probabilities for each fragmentation pattern model. 2) Healthy individuals were selected from the Cristiano et al. dataset and fragmentation pattern vectors for each healthy sample were computed. The median value across all healthy samples was obtained as the fragmentation pattern value for that particular region. Analyze the Spearman correlation between vectors composed of the median values of fragmentation patterns (consistent dimensions were required for Spearman correlation analysis; hence, only 6 fragmentation patterns with consistent dimensions were used).

### Important Open Chromatin Region Screening

In the classifier model, the absolute values of feature parameters were used to evaluate feature weights during model training. In the ensemble classifier, each open chromatin region had 7 weight parameters (one for each fragmentation pattern). As the length, FSD, and EDM lacked specific information for each interval, parameters of the other 7 fragmentation patterns were used. The sum of absolute values was calculated as a measure of the importance of each open chromatin region. The top 15k regions (features) with the highest rankings were selected as important open chromatin regions. An integrated classifier was utilized for BRCA to interpret the biological functions of the selected features.

### Gene Mapping and Functional Annotation Analysis

The top 15k regions with the highest contribution importance to the BRCA diagnostic model were considered. For gene mapping, the Cistrome‐GO analysis tool enhancer mode was used, with all the top 15k regions. Functional annotation of the Kyoto Encyclopedia of Genes and Genomes pathways and gene ontology was performed using Cistrome‐GO with default parameters.^[^ ^65^ ^]^

### The IFS.CvN.Low‐Sig Signature

The signature of genes corresponding to regions with significantly lower IFS scores in patients with BRCA than in controls, named IFS.CvN.low‐sig was constructed. The Wilcoxon rank‐sum test was used for systematic comparisons; 585 regions with a lower mean IFS score in patients with BRCA than that in controls and a *p*‐value < 0.01 were included. A gene‐mapping approach similar to that used in the Cistrome‐GO enhancer mode was applied,^[^ ^65^ ^]^ resulting in a total of 1431 protein‐coding genes that comprised the signature.

### Bulk Transcriptomic Analysis

RNA‐seq transcriptomes (log2 RSEM) of samples included in the TCGA BRCA project, including 1104 BRCA tumors and 114 adjacent normal tissues are shown in Table S1 (Supporting Information). The IFS.CvN.low‐sig meta‐gene expression was calculated using gene set variation analysis. Wilcoxon rank‐sum test was performed to test the statistical significance in IFS.CvN.low‐sig meta‐gene expression between BRCA tumors and adjacent normal tissues.

### Single‐Cell RNA‐Seq Analysis

Single‐cell RNA‐seq analysis was performed as previously described.^[^ ^87^ ^]^ Briefly, publicly available data GSE176078 were downloaded from the Gene Expression Omnibus database (Table S1, Supporting Information). Seurat R package was used for subsequent analyses. Lineage‐level cell types, including epithelial, fibroblast, endothelial, pericyte, myeloid, T, and B cells were annotated via clustering after quality control and cell filtering. Meta‐gene expression was calculated using the AddModuleScore function and AUCell algorithm.^[^ ^88^ ^]^

### Tumor DNA Fraction Estimation

The tumor DNA fraction of each cfDNA sample was estimated using ichorCNA v0.2.04.^[^ ^64^ ^]^ by employing the same parameters as in the previous study.^[^ ^10^ ^]^

### Statistical Analysis

Before constructing the classification models, cfDNA fragmentation patterns were normalized to Z‐scores. The figure data were presented as means and 95% confidence intervals. For differential analysis of the 2 data groups (unpaired), the Wilcoxon rank‐sum test was used. The Kruskal‐Wallis test was used for the differential analysis of multiple data groups (unpaired). For all statistical models, two‐tailed tests were used, and a *p*‐value < 0.05 was considered statistically significant. All statistical models were implemented in R4.1.2. Fragmentation patterns and machine learning model construction were calculated using Python3.8.17.

## Conflict of Interest

The authors declare no conflict of interest.

## Author Contributions

X.Z. and X.M. conceived the study. X.Z. and Y.H. designed the methodological framework. Y.H. and X.M. performed the data analysis. Y.H., X.Z., and X.M. wrote the manuscript together. All the authors read and approved the final manuscript.

## Supporting information

Supporting Information

Supporting Information

## Data Availability

The data that support the findings of this study are available from the corresponding author upon reasonable request.
