# Supplementary material for: Systematically Evaluating Cell‐Free DNA Fragmentation Patterns for Cancer Diagnosis and Enhanced Cancer Detection via Integrating Multiple Fragmentation Patterns
Source: Adv Sci (Weinh). 2024 Jun 17;11(30):2308243. doi: 10.1002/advs.202308243 (PMC11321639; doi:10.1002/advs.202308243)
Supplement: Supplementary file 1 — Supporting Information [file ADVS-11-2308243-s002.docx]

Systematically Evaluating Cell-Free DNA Fragmentation Patterns for Cancer Diagnosis and Enhanced Cancer Detection via Integrating Multiple Fragmentation Patterns

Yuying Hou^1^, Xiang-Yu Meng^1,3^*, Xionghui Zhou^1,2^*

^1^Hubei Key Laboratory of Agricultural Bioinformatics, College of Informatics, Huazhong Agricultural University, Wuhan 430070, People’s Republic of China

^2^Key Laboratory of Smart Farming for Agricultural Animals, Ministry of Agriculture and Rural Affairs, People’s Republic of China

^3^Health Science Center, Hubei Minzu University, Enshi 445000, People’s Republic of China

*Correspondence: mengxy_whu@163.com (X.M); zhouxionghui@mail.hzau.edu.cn (X.Z.)

**Supplementary Materials for**

Systematically Evaluating Cell-Free DNA Fragmentation Patterns for Cancer Diagnosis and Enhanced Cancer Detection via Integrating Multiple Fragmentation Patterns

This file includes:

Supplementary Figure S1-6

Figure S1. Performance of 10 cfDNA fragmentation patterns using cross-validation in the Cristiano et al. and Jiang et al. datasets.

Figure S2. Spearman correlation matrix among cfDNA fragmentation patterns.

Figure S3. Comparison of the performance of Integrated Fragmentation Pattern (IFP) and 10 cfDNA fragmentation patterns.

Figure S4. Actual types and second-ranked predicted types for each cancer in the multiclass model constructed using the Integrated Fragmentation Pattern (IFP) from the Cristiano et al. dataset (excluding lung cancer).

Figure S5. Results of multiclass classification using the Integrated Fragmentation Pattern (IFP) from the Cristiano et al. dataset (including lung cancer).

Figure S6. Comparing the classification results of using five machine learning models to build classification models for all cfDNA fragmentation patterns in the Cristiano et al. dataset.

**
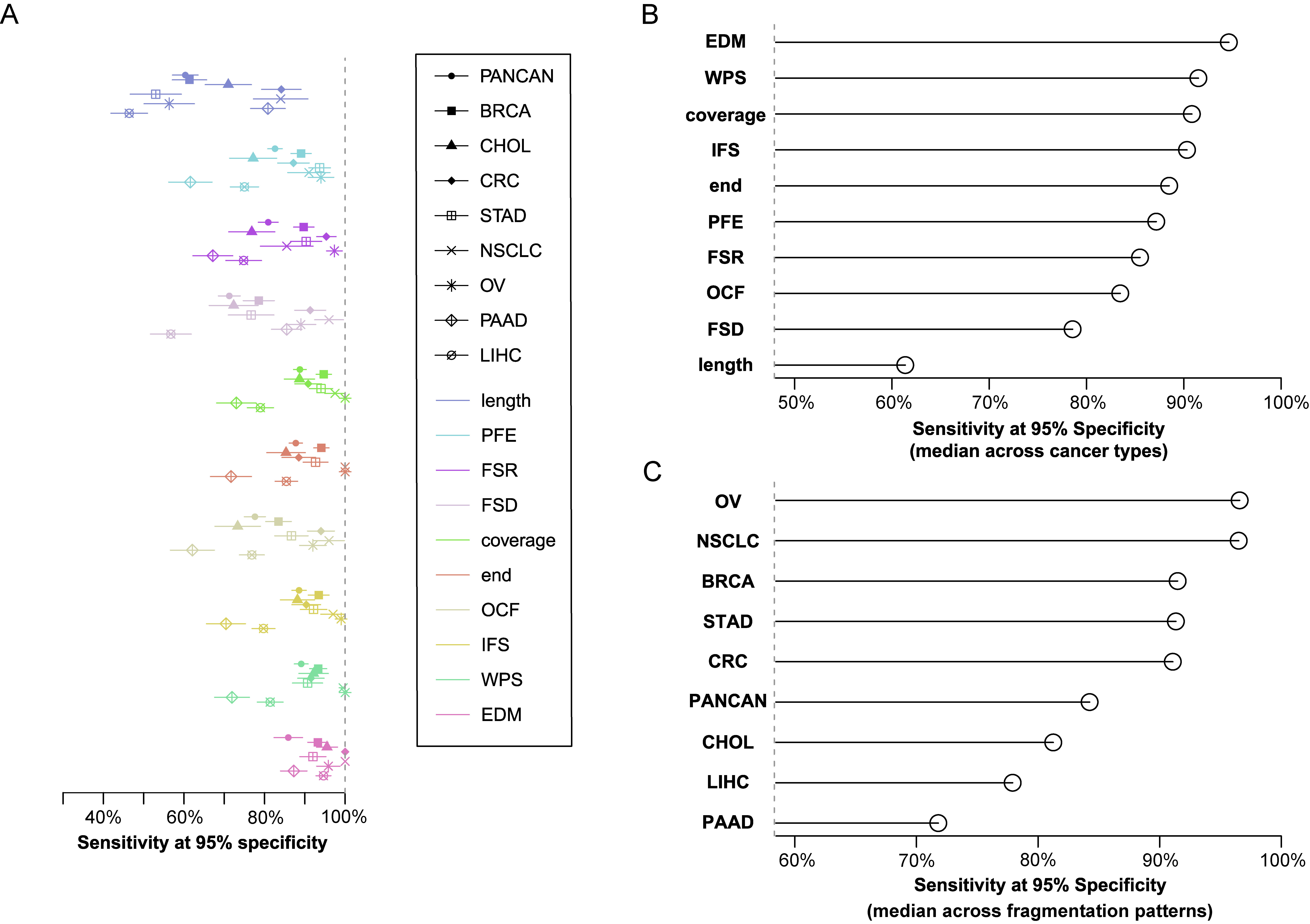
**

**Figure S1. Performance of 10 cfDNA fragmentation patterns using cross-validation in the Cristiano et al. and Jiang et al. datasets.** (A) Performance of all cfDNA fragmentation patterns across all cancer types (sensitivity at 95% specificity). The data are presented as means and 95% confidence intervals. (B) Ranking of median values of each cfDNA fragmentation pattern across all cancer types (sensitivity at 95% specificity). (C) Ranking of median values of each cancer type across all cfDNA fragmentation patterns (sensitivity at 95% specificity).


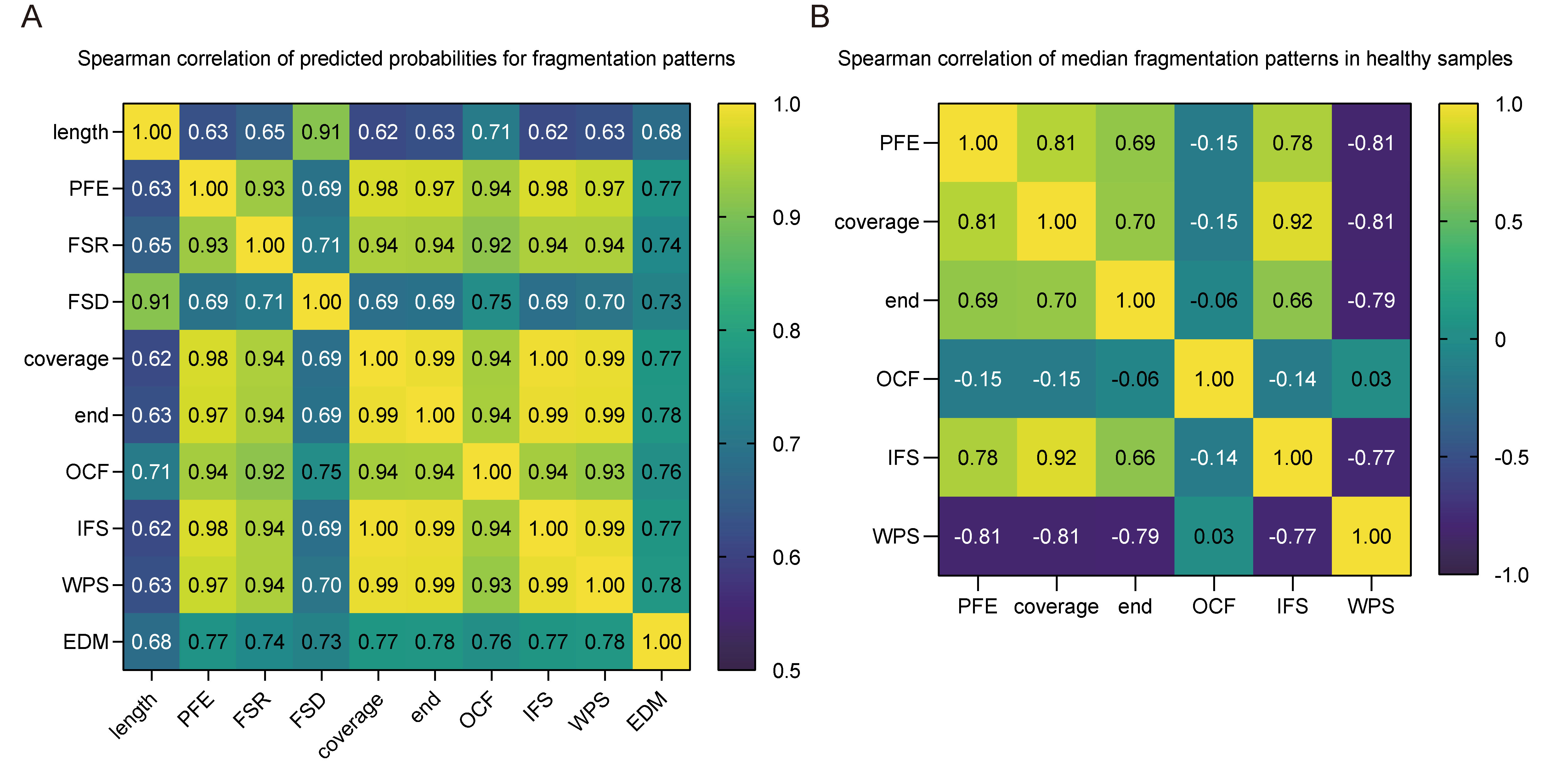


**Figure S2. Spearman correlation matrix among cfDNA fragmentation patterns.** (A) Construct cancer diagnostic models using each cfDNA fragmentation pattern separately in the Cristiano et al. dataset. Output the model’s predicted probabilities for each sample and analyze the Spearman correlation between these predicted probabilities. (B) Utilizing healthy samples from the Cristiano et al. dataset., calculate the median of each cfDNA fragmentation pattern separately. Analyze the Spearman correlation between the medians of different fragmentation patterns (Due to the requirement of consistent dimensions for Spearman correlation analysis, only six fragmentation patterns with consistent dimensions were used in this part).


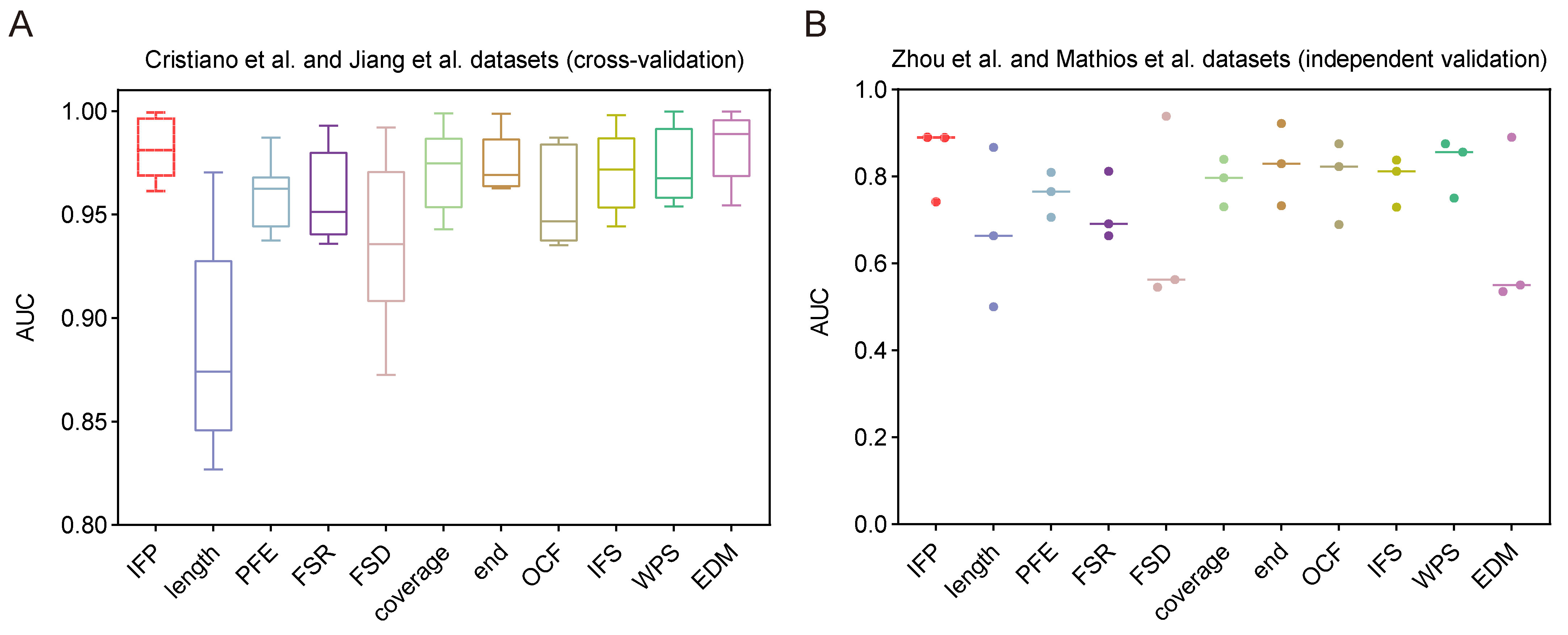


**Figure S3. Comparison of the performance of Integrated Fragmentation Pattern (IFP) and 10 cfDNA fragmentation patterns.** (A) Cross-validation on Cristiano et al. and Jiang et al. datasets. (B) Independent validation on Zhou et al. and Mathios et al. datasets.

**
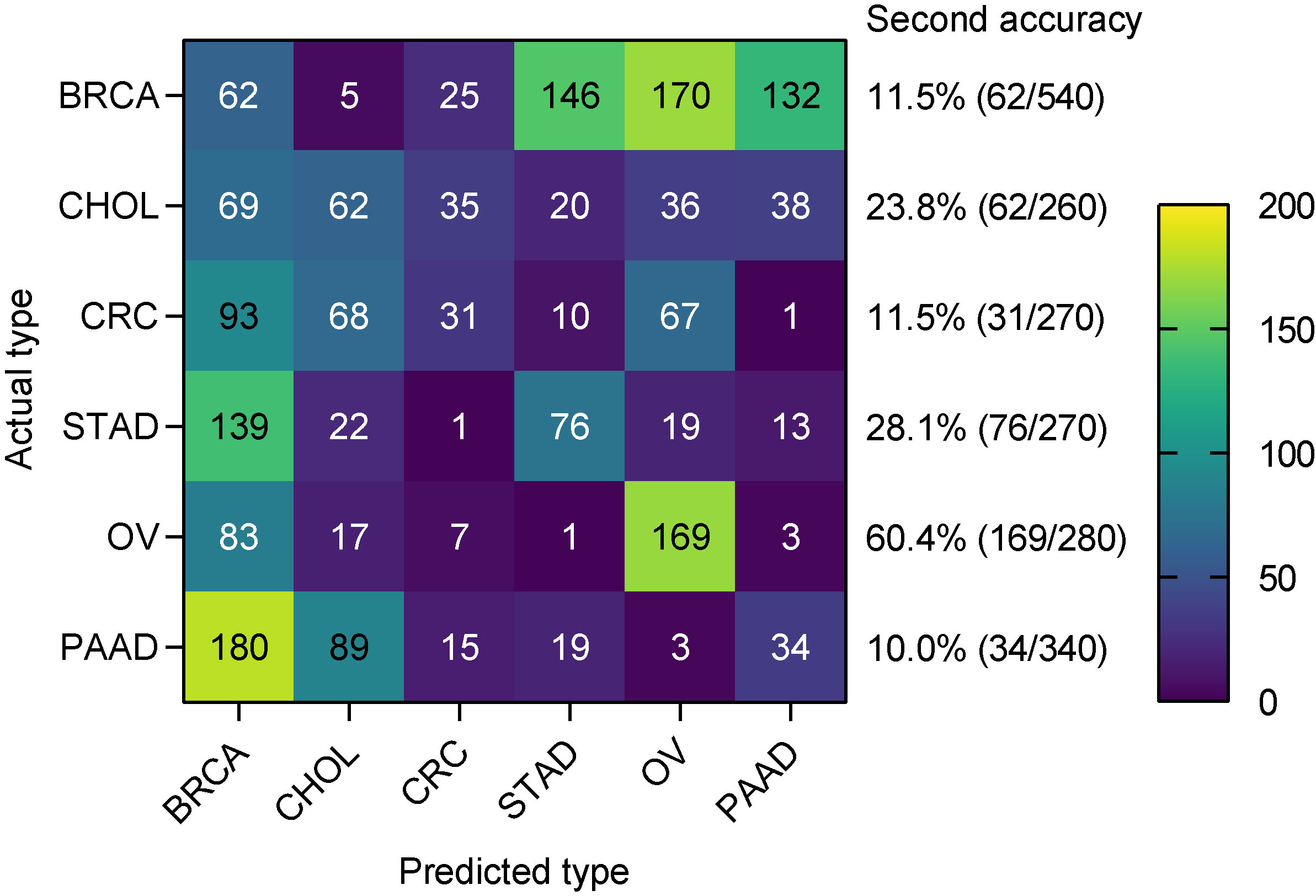
**

**Figure S4. Actual types and second-ranked predicted types for each cancer in the multiclass model constructed using the Integrated Fragmentation Pattern (IFP) from the Cristiano et al. dataset (excluding lung cancer).**


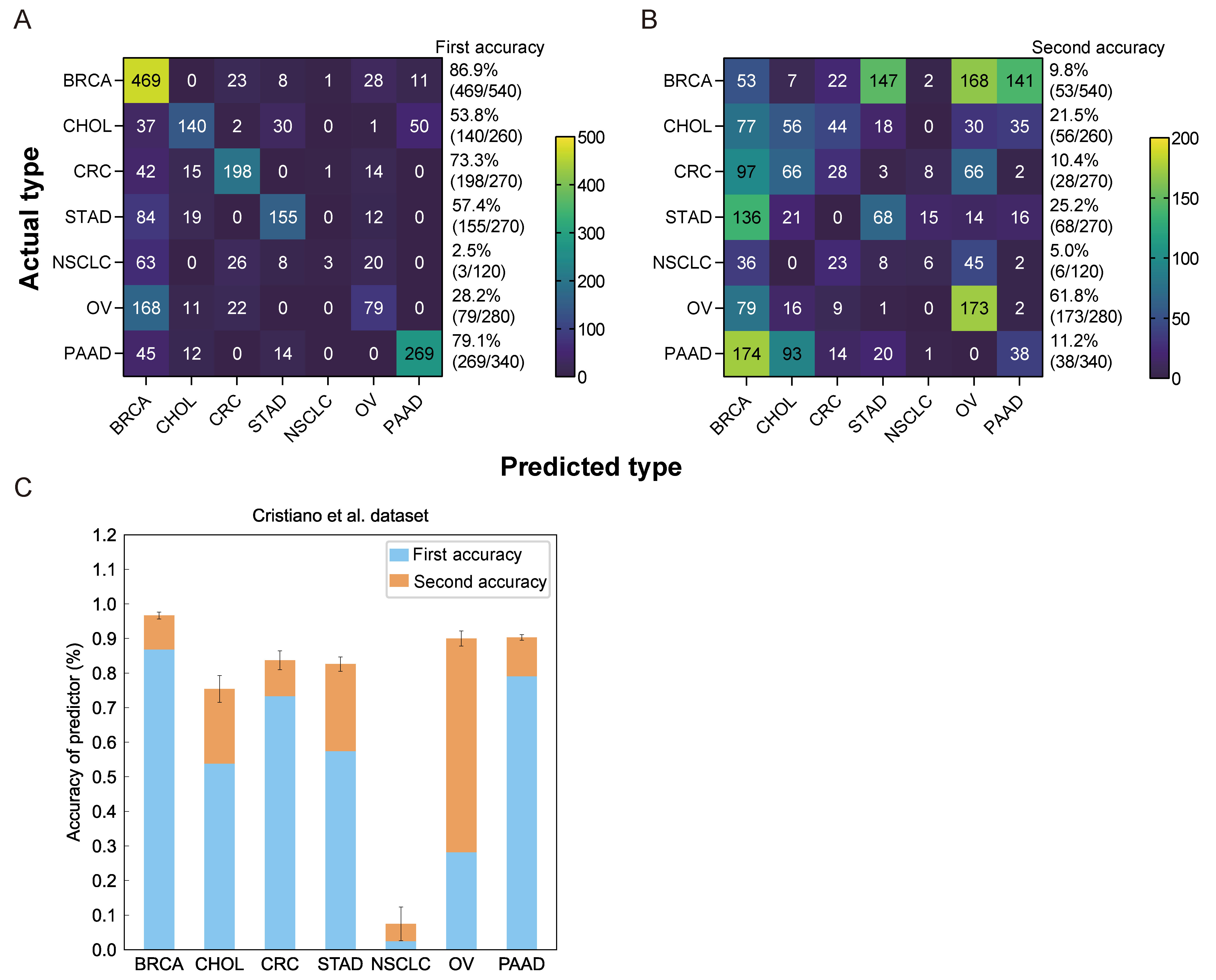


**Figure S5. Results of multiclass classification using the Integrated Fragmentation Pattern (IFP) from the Cristiano et al. dataset (including lung cancer).** (A) The actual type and first-ranked predicted type for each cancer in the multiclass model. (B) The actual type and second-ranked predicted type for each cancer in the multiclass model. (C) The first and second accuracy for each cancer in the multiclass model. The data are presented as means and 95% confidence intervals.

**
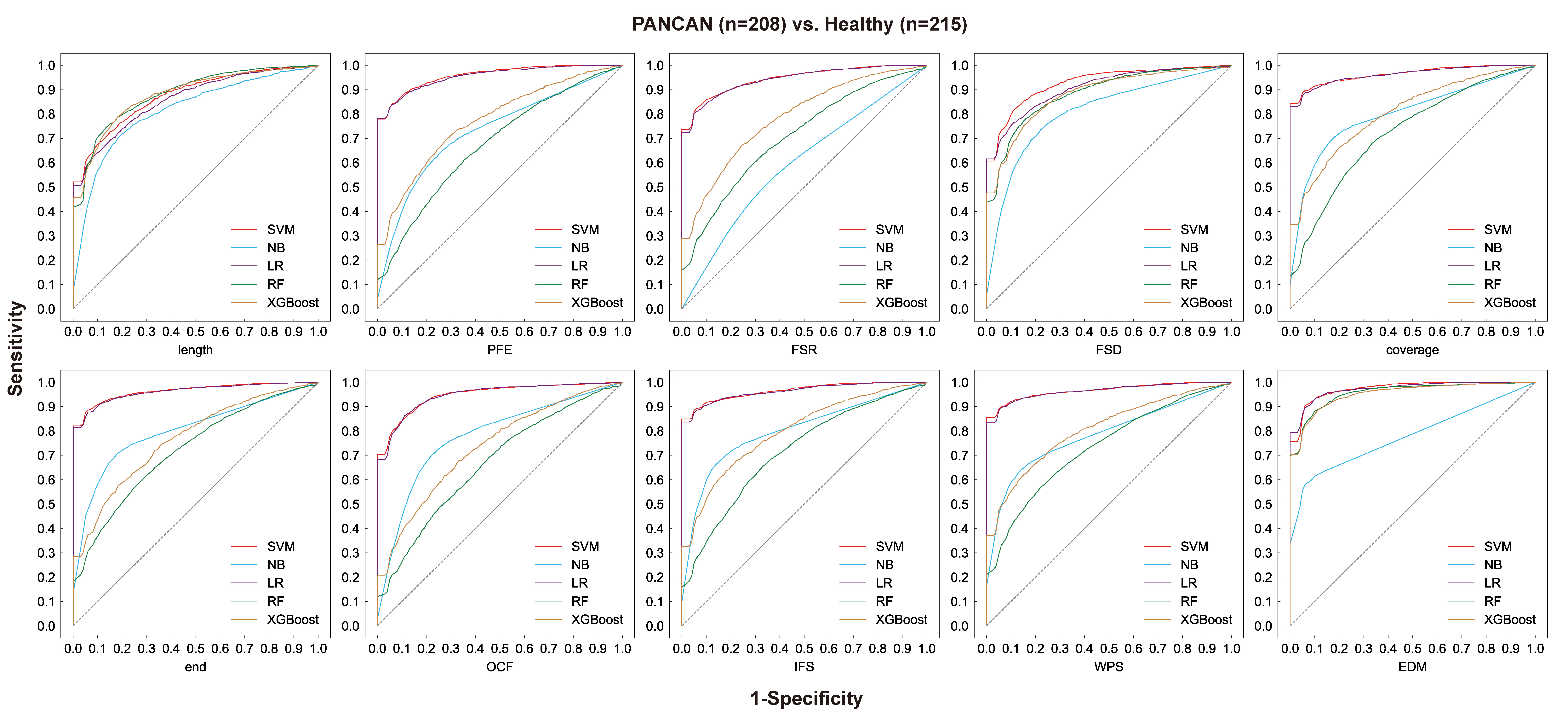
Figure S6. Comparing the classification results of using five machine learning models to build classification models for all cfDNA fragmentation patterns in the Cristiano et al. dataset.** SVM: Support Vector Machine; NB: Naïve Bayes; LR: Logistic Regression; RF: Random Forest.
